# *trans*-Di-μ-chlorido-bis­{chlorido[tris­(3,5-dimethyl­phen­yl)phosphane-κ*P*]palla­dium(II)} dichloro­methane monosolvate

**DOI:** 10.1107/S1600536812048556

**Published:** 2012-11-30

**Authors:** Wade L. Davis, Alfred Muller

**Affiliations:** a Research Centre for Synthesis and Catalysis, Department of Chemistry, University of Johannesburg (APK Campus), PO Box 524, Auckland Park, Johannesburg, 2006, South Africa

## Abstract

In the dimeric title compound, [Pd_2_Cl_4_{P(C_8_H_9_)_3_}_2_]·CH_2_Cl_2_, the metal complex molecule is situated about an inversion centre and is accompanied by a dichloro­methane solvent mol­ecule situated on a twofold rotation axis. The Pd^II^ atom has a slightly distorted square-planar coordination sphere. The effective cone angle for the tris­(3,5-dimethyl­phen­yl)phos­phane ligand was calculated to be 169°. In the crystal, the metal complex and solvent mol­ecules are linked *via* C—H⋯Cl inter­actions, generating chains along [10-2]. There are also C—H⋯π and weak π–π inter­actions present [centroid–centroid distance = 3.990 (2) Å, plane–plane distance = 3.6352 (15) Å and ring slippage = 1.644 Å], forming of a three-dimensional structure.

## Related literature

For background on catalysis of palladium compounds, see: Bedford *et al.* (2004 ▶). For the synthesis of the starting materials, see: Drew & Doyle (1990 ▶). For a description of the Cambridge Structural Database, see: Allen (2002 ▶). For background on cone angles, see: Tolman (1977 ▶); Otto (2001 ▶).
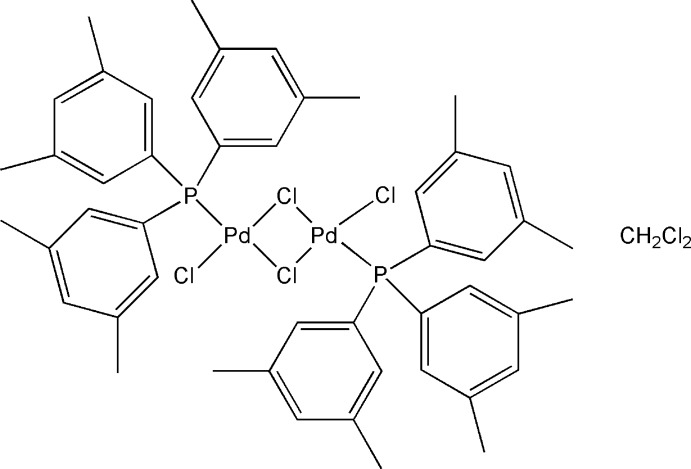


## Experimental

### 

#### Crystal data

[Pd_2_Cl_4_(C_24_H_27_P)_2_]·CH_2_Cl_2_
*M* *_r_* = 1132.38
Monoclinic, 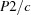

*a* = 14.747 (2) Å
*b* = 9.1038 (13) Å
*c* = 21.376 (3) Å
β = 117.576 (8)°
*V* = 2543.8 (6) Å^3^
*Z* = 2
Mo *K*α radiation
μ = 1.12 mm^−1^
*T* = 100 K
0.19 × 0.16 × 0.13 mm

#### Data collection

Bruker APEX DUO 4K-CCD diffractometer
Absorption correction: multi-scan (*SADABS*; Bruker, 2008 ▶) *T*_min_ = 0.816, *T*_max_ = 0.868
30952 measured reflections
6349 independent reflections
4776 reflections with *I* > 2σ(*I*)
*R*_int_ = 0.071

#### Refinement

*R*[*F* ^2^ > 2σ(*F* ^2^)] = 0.040
*wR*(*F* ^2^) = 0.098
*S* = 1.03
6349 reflections
273 parameters
H-atom parameters constrained
Δρ_max_ = 0.90 e Å^−3^
Δρ_min_ = −1.12 e Å^−3^

### 

Data collection: *APEX2* (Bruker, 2011 ▶); cell refinement: *SAINT* (Bruker, 2008 ▶); data reduction: *SAINT* and *XPREP* (Bruker, 2008 ▶); program(s) used to solve structure: *SIR97* (Altomare *et al.*, 1999 ▶); program(s) used to refine structure: *SHELXL97* (Sheldrick, 2008 ▶); molecular graphics: *DIAMOND* (Brandenburg & Putz, 2005 ▶); software used to prepare material for publication: *publCIF* (Westrip, 2010 ▶) and *WinGX* (Farrugia, 2012 ▶).

## Supplementary Material

Crystal structure: contains datablock(s) global, I. DOI: 10.1107/S1600536812048556/su2534sup1.cif

Structure factors: contains datablock(s) I. DOI: 10.1107/S1600536812048556/su2534Isup2.hkl

Additional supplementary materials:  crystallographic information; 3D view; checkCIF report

## Figures and Tables

**Table 1:** Hydrogen-bond geometry (Å, °) *Cg*1 and *Cg*2 are the centroids of rings C17–C19/C21/C22/C24 and C9–C11/C13/C14/C16, respectively.

*D*—H⋯*A*	*D*—H	H⋯*A*	*D*⋯*A*	*D*—H⋯*A*
C25—H25*A*⋯Cl2	0.99	2.82	3.733 (4)	154
C21—H21⋯Cl1^i^	0.95	2.85	3.693 (4)	148
C5—H5⋯*Cg*1^ii^	0.95	2.95	3.847 (5)	159
C15—H15*A*⋯*Cg*2^iii^	0.99	2.79	3.620 (5)	143

Symmetry codes: (i) 
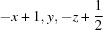
; (ii) 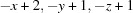; (iii) 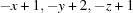.

**Fig. 1.**
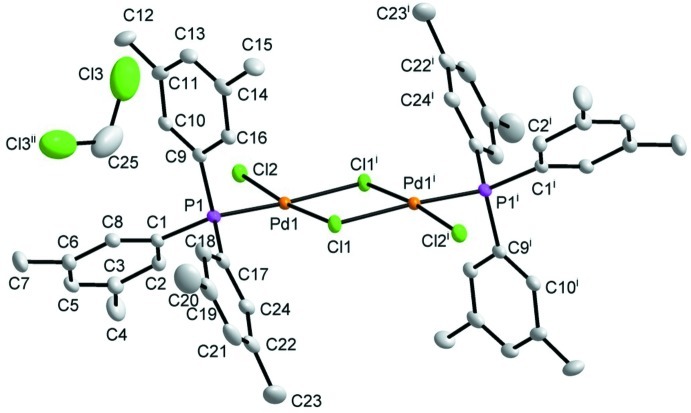
A view of the molecular structure of the title complex, showing the atom-numbering. Displacement ellipsoids are drawn at the 50% probability level. [symmetry code (i) = -x+1, -y+1, -z+1; H atoms have been omitted for clarity].

**Fig. 2.**
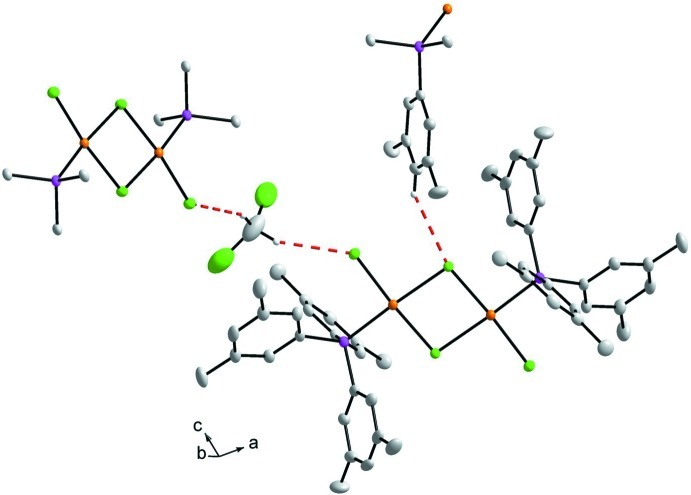
A view of the crystal packing of the title compound, showing the C—H···Cl interactions (red dashed lines) between the metal complex and the dichloromethane solvate. H atoms not involved in H-bonding have been omitted for clarity.

**Fig. 3.**
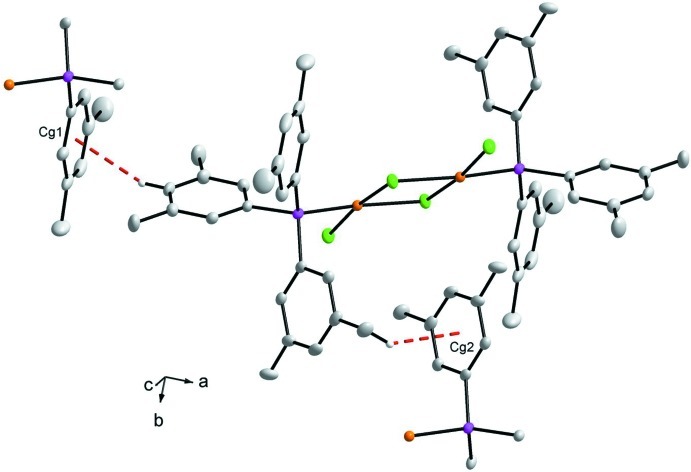
A view of the crystal packing of the title compound, showing the C—H···π interactions (red dashed lines). H atoms not involved in H-bonding have been omitted for clarity.

**Fig. 4.**
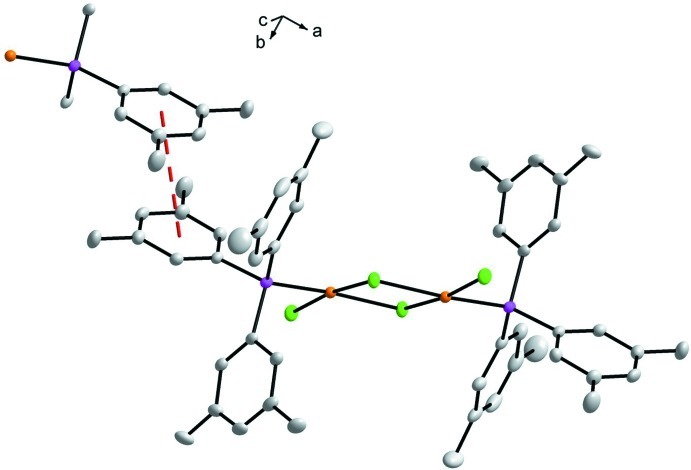
A view of the crystal packing of the title compound, showing the π···π interactions (red dashed lines). H atoms have been omitted for clarity.

## supplementary crystallographic information

### Comment

Complexes involving palladium metal centres are among some of the most popular catalytic precursors in organic synthesis due to their catalytic abilities. They are used in carbon-carbon bond formation reactions, *e.g.* the Heck, Stille and Suzuki reactions (Bedford *et al.*, 2004). [PdCl_2_(*L*)_2_] (*L* = tertiary phosphine, arsine or stibine) complexes can conveniently be prepared by the substitution of 1,5-cyclooctadiene (COD) from [PdCl_2_(COD)]. The title compound is the product of the reaction of [PdCl_2_(COD)] with tris(3,5-dimethylphenyl)phosphane as ligand, which shows dimerization of the square-planar Pd^II^ monomer. The crystal structure reported on herein is, to the best of our knowledge, the first Pd complex containing this phosphane ligand.

In the title compound, Fig. 1, the dimeric Pd^II^ complex is situated about an inversion centre and crystallizes with a dichloromethane solvate molecule that is located on a 2-fold rotation axis. Each equivalent pair of terminal bonded ligands is in a mutually *trans* orientation, with only slight distortions in the P1—Pd1—Cl1 and Cl2—Pd1—Cl1 angles of 173.90 (3) and 173.20 (3)°, respectively. The distortion of the square-planar metal coordination is further exemplified by the displacement of the Pd^II^ metal centre by 0.1122 (4) Å from the plane formed by the coordinating atoms Cl2/P1/Cl1/Cl1^i^ (symmetry code: (i) = -x+1, -y+1, -z+1; r.m.s. deviation of mean plane = 0.0085 Å).

To describe the steric demand of the phosphane ligand the Tolman cone angle (Tolman, 1977) is still the most commonly used model. Applying this model to the geometry obtained for the title compound (and adjusting the Pd—P bond distance to 2.28 Å) we calculated an effective cone angle (Otto, 2001) of 169°. A search of the Cambridge Structural Database (CSD, V5.33, last update Aug. 2012; Allen, 2002) gave only three hits for structures containing the tris(3,5-dimethylphenyl)phosphane moiety. Cone angle calculations for these structures gave values ranging from 160 to 180°, with the value obtained for the title compound (169°) fitting well in this range.

In the crystal, weak C—H···Cl interactions between the dichloromethane solvate and the dimeric metal complex generate chains along the [1 0 -2] direction (Fig. 2 and Table 1). Additionally, several C—H···π (Fig. 3 and Table 1) and π-π stacking interactions (centroid-to-centroid distance = 3.990 (2) Å, plane-to plane separation 3.6352 (15) Å, ring slippage = 1.644 Å) are observed (Fig. 4), leading to the formation of a three-dimensional structure.

### Experimental

Dichloro(1,5-cyclooctadiene)palladium(II), [PdCl_2_(COD)], was prepared according to the literature procedure of Drew & Doyle (1990). Tris(3,5-dimethylphenyl)phosphane (12.1 mg, 0.035 mmol) was dissolved in CH_2_Cl_2_ (5 cm^3^). A solution of [Pd(COD)Cl_2_] (5.0 mg, 0.017 mmol) in CH_2_Cl_2_ (5 cm^3^) was added to the phosphane solution. The mixture was stirred for 2hr at room temperature, after which the solution was left to slowly evaporate. Dark red crystals of the title compound suitable for a single-crystal X-ray study were obtained. Spectroscopic data for the title compound are available in the archived CIF.

### Refinement

The H atoms were placed in calculated positions and allowed to ride on their parent atoms: C—H = 0.95, 0.99 and 0.98 Å for CH, CH_2_ and CH_3_ H atoms, respectively, with *U*_iso_(H) = k × *U*_eq_(C), where k = 1.5 for methyl H atoms and = 1.2 for other H atoms. Methyl torsion angles were refined from electron density. The deepest residual electron-density hole (-1.12 eÅ^3^) is located at 0.71 Å from Cl3 and the highest peak (0.9 eÅ^3^) 0.86 Å from Pd1.

### Crystal data

**Table:**

[Pd_2_Cl_4_(C_24_H_27_P)_2_]·CH_2_Cl_2_	*F*(000) = 1148
*M**_r_* = 1132.38	*D*_x_ = 1.478 Mg m^−^^3^
Monoclinic, *P*2/*c*	Mo *K*α radiation, λ = 0.71073 Å
Hall symbol: -P 2yc	Cell parameters from 4452 reflections
*a* = 14.747 (2) Å	θ = 2.2–27.5°
*b* = 9.1038 (13) Å	µ = 1.12 mm^−^^1^
*c* = 21.376 (3) Å	*T* = 100 K
β = 117.576 (8)°	Cube, orange
*V* = 2543.8 (6) Å^3^	0.19 × 0.16 × 0.13 mm
*Z* = 2	

### Data collection

**Table:**

Bruker APEX DUO 4K-CCD diffractometer	6349 independent reflections
Radiation source: sealed tube	4776 reflections with *I* > 2σ(*I*)
Graphite monochromator	*R*_int_ = 0.071
Detector resolution: 8.4 pixels mm^-1^	θ_max_ = 28.4°, θ_min_ = 1.6°
φ and ω scans	*h* = −19→19
Absorption correction: multi-scan (*SADABS*; Bruker, 2008)	*k* = −12→12
*T*_min_ = 0.816, *T*_max_ = 0.868	*l* = −28→28
30952 measured reflections	

### Refinement

**Table:**

Refinement on *F*^2^	Primary atom site location: structure-invariant direct methods
Least-squares matrix: full	Secondary atom site location: difference Fourier map
*R*[*F*^2^ > 2σ(*F*^2^)] = 0.040	Hydrogen site location: inferred from neighbouring sites
*wR*(*F*^2^) = 0.098	H-atom parameters constrained
*S* = 1.03	*w* = 1/[σ^2^(*F*_o_^2^) + (0.0419*P*)^2^ + 1.7351*P*] where *P* = (*F*_o_^2^ + 2*F*_c_^2^)/3
6349 reflections	(Δ/σ)_max_ = 0.001
273 parameters	Δρ_max_ = 0.90 e Å^−^^3^
0 restraints	Δρ_min_ = −1.12 e Å^−^^3^

### Special details

**Table:**

Experimental. Spectroscopic data for the title compund: ^31^P NMR (CDCl_3_, 162.0 MHz): δ (p.p.m.) 33.54 (s, 1P). ^1^H NMR (CDCl_3_, 400 MHz): δ (p.p.m.) 2.34 (m, 36H), 7.11 (m, 4H), 7.34 (m, 4H), 7.36 (m, 2H) 7.66 (m, 6H), 7.32 (m, 4H).
Geometry. All e.s.d.'s (except the e.s.d. in the dihedral angle between two l.s. planes) are estimated using the full covariance matrix. The cell e.s.d.'s are taken into account individually in the estimation of e.s.d.'s in distances, angles and torsion angles; correlations between e.s.d.'s in cell parameters are only used when they are defined by crystal symmetry. An approximate (isotropic) treatment of cell e.s.d.'s is used for estimating e.s.d.'s involving l.s. planes.
Refinement. Refinement of *F*^2^ against ALL reflections. The weighted *R*-factor *wR* and goodness of fit *S* are based on *F*^2^, conventional *R*-factors *R* are based on *F*, with *F* set to zero for negative *F*^2^. The threshold expression of *F*^2^ > σ(*F*^2^) is used only for calculating *R*-factors(gt) *etc*. and is not relevant to the choice of reflections for refinement. *R*-factors based on *F*^2^ are statistically about twice as large as those based on *F*, and *R*- factors based on ALL data will be even larger.

### Fractional atomic coordinates and isotropic or equivalent isotropic displacement parameters (Å^2^)

**Table:**

	*x*	*y*	*z*	*U*_iso_*/*U*_eq_	Occ. (<1)
Pd1	0.627522 (18)	0.56103 (3)	0.537254 (13)	0.01368 (8)	
Cl1	0.49039 (6)	0.53788 (9)	0.42440 (4)	0.01841 (17)	
Cl2	0.74975 (6)	0.60182 (10)	0.65077 (4)	0.02141 (19)	
P1	0.72374 (6)	0.67044 (10)	0.49592 (4)	0.01491 (18)	
C1	0.8616 (2)	0.6459 (4)	0.53793 (17)	0.0175 (7)	
C2	0.9098 (3)	0.5328 (4)	0.58601 (18)	0.0214 (7)	
H2	0.8715	0.472	0.6012	0.026*	
C3	1.0148 (3)	0.5092 (4)	0.6118 (2)	0.0258 (8)	
C4	1.0691 (3)	0.3890 (5)	0.6654 (2)	0.0391 (11)	
H4A	1.0903	0.311	0.6434	0.059*	
H4B	1.0226	0.3482	0.682	0.059*	
H4C	1.1295	0.43	0.7055	0.059*	
C5	1.0682 (3)	0.6006 (4)	0.5881 (2)	0.0268 (9)	
H5	1.1393	0.5843	0.6051	0.032*	
C6	1.0219 (3)	0.7144 (4)	0.54062 (19)	0.0245 (8)	
C7	1.0833 (3)	0.8123 (5)	0.5172 (2)	0.0371 (10)	
H7A	1.1272	0.7517	0.5045	0.056*	
H7B	1.1258	0.8788	0.5558	0.056*	
H7C	1.0367	0.87	0.4761	0.056*	
C8	0.9174 (2)	0.7360 (4)	0.51530 (18)	0.0202 (7)	
H8	0.8837	0.8125	0.4824	0.024*	
C9	0.6976 (2)	0.8629 (4)	0.49952 (18)	0.0174 (7)	
C10	0.7633 (2)	0.9478 (4)	0.55679 (18)	0.0187 (7)	
H10	0.8275	0.909	0.5899	0.022*	
C11	0.7352 (3)	1.0906 (4)	0.56585 (19)	0.0224 (8)	
C12	0.8050 (3)	1.1774 (4)	0.6300 (2)	0.0315 (9)	
H12A	0.8125	1.1266	0.6725	0.047*	
H12B	0.7758	1.2752	0.6277	0.047*	
H12C	0.8722	1.1871	0.6315	0.047*	
C13	0.6423 (3)	1.1449 (4)	0.5154 (2)	0.0234 (8)	
H13	0.6236	1.2422	0.5207	0.028*	
C14	0.5749 (3)	1.0627 (4)	0.45713 (19)	0.0228 (8)	
C15	0.4734 (3)	1.1257 (4)	0.4041 (2)	0.0280 (8)	
H15A	0.4296	1.1409	0.4267	0.042*	
H15B	0.4401	1.0573	0.3645	0.042*	
H15C	0.4848	1.2198	0.3866	0.042*	
C16	0.6032 (3)	0.9208 (4)	0.45022 (19)	0.0199 (7)	
H16	0.5582	0.862	0.4116	0.024*	
C17	0.6861 (2)	0.6207 (4)	0.40528 (17)	0.0196 (7)	
C18	0.6758 (3)	0.7236 (4)	0.35443 (18)	0.0244 (8)	
H18	0.6843	0.8251	0.3661	0.029*	
C19	0.6527 (3)	0.6785 (5)	0.28588 (19)	0.0305 (9)	
C20	0.6411 (4)	0.7934 (6)	0.2318 (2)	0.0491 (13)	
H20A	0.5764	0.8459	0.217	0.074*	
H20B	0.6413	0.7457	0.1907	0.074*	
H20C	0.6981	0.863	0.2525	0.074*	
C21	0.6429 (3)	0.5291 (5)	0.2705 (2)	0.0338 (10)	
H21	0.6283	0.4982	0.2243	0.041*	
C22	0.6539 (3)	0.4227 (5)	0.3208 (2)	0.0286 (9)	
C23	0.6476 (3)	0.2622 (5)	0.3036 (2)	0.0421 (11)	
H23A	0.6304	0.2069	0.336	0.063*	
H23B	0.7137	0.2285	0.3085	0.063*	
H23C	0.5946	0.2462	0.2549	0.063*	
C24	0.6751 (2)	0.4700 (4)	0.38800 (19)	0.0228 (8)	
H24	0.6822	0.3997	0.4229	0.027*	
Cl3	0.94842 (13)	0.9023 (2)	0.79374 (9)	0.0900 (6)	
C25	1	0.7942 (9)	0.75	0.075 (3)	
H25A	0.9457	0.7302	0.7153	0.09*	0.5
H25B	1.0543	0.7302	0.7847	0.09*	0.5

### Atomic displacement parameters (Å^2^)

**Table:**

	*U*^11^	*U*^22^	*U*^33^	*U*^12^	*U*^13^	*U*^23^
Pd1	0.01213 (12)	0.01374 (13)	0.01494 (13)	0.00036 (10)	0.00608 (9)	−0.00087 (10)
Cl1	0.0153 (4)	0.0246 (4)	0.0149 (4)	−0.0037 (3)	0.0066 (3)	−0.0005 (3)
Cl2	0.0167 (4)	0.0287 (5)	0.0168 (4)	−0.0024 (3)	0.0061 (3)	−0.0026 (3)
P1	0.0136 (4)	0.0145 (4)	0.0174 (4)	0.0015 (3)	0.0077 (3)	−0.0005 (3)
C1	0.0139 (15)	0.0186 (18)	0.0211 (17)	0.0003 (13)	0.0089 (14)	−0.0036 (14)
C2	0.0171 (16)	0.0199 (19)	0.0270 (18)	−0.0014 (13)	0.0101 (15)	−0.0013 (15)
C3	0.0186 (17)	0.024 (2)	0.032 (2)	0.0030 (15)	0.0097 (16)	−0.0002 (17)
C4	0.0206 (19)	0.042 (3)	0.049 (3)	0.0067 (18)	0.0111 (19)	0.010 (2)
C5	0.0147 (16)	0.029 (2)	0.037 (2)	0.0013 (14)	0.0121 (16)	−0.0086 (17)
C6	0.0190 (17)	0.025 (2)	0.031 (2)	−0.0029 (14)	0.0133 (16)	−0.0064 (16)
C7	0.025 (2)	0.033 (2)	0.062 (3)	−0.0014 (17)	0.027 (2)	−0.001 (2)
C8	0.0187 (17)	0.0180 (18)	0.0257 (18)	0.0014 (13)	0.0119 (15)	−0.0018 (15)
C9	0.0187 (16)	0.0142 (17)	0.0248 (17)	0.0012 (13)	0.0147 (14)	0.0018 (14)
C10	0.0170 (15)	0.0184 (17)	0.0245 (17)	0.0022 (14)	0.0129 (14)	0.0022 (15)
C11	0.0299 (19)	0.0149 (18)	0.0302 (19)	−0.0010 (14)	0.0206 (17)	−0.0012 (14)
C12	0.040 (2)	0.017 (2)	0.038 (2)	−0.0007 (17)	0.0180 (19)	−0.0064 (17)
C13	0.0294 (19)	0.0130 (17)	0.035 (2)	0.0037 (14)	0.0212 (17)	0.0038 (15)
C14	0.0248 (18)	0.0220 (18)	0.0297 (19)	0.0069 (15)	0.0194 (16)	0.0087 (16)
C15	0.029 (2)	0.022 (2)	0.034 (2)	0.0102 (16)	0.0150 (17)	0.0080 (17)
C16	0.0183 (16)	0.0172 (18)	0.0272 (18)	0.0019 (13)	0.0131 (15)	0.0026 (15)
C17	0.0122 (15)	0.028 (2)	0.0191 (17)	0.0012 (14)	0.0078 (14)	−0.0027 (15)
C18	0.0187 (17)	0.033 (2)	0.0215 (18)	0.0006 (15)	0.0097 (15)	0.0018 (16)
C19	0.0182 (18)	0.054 (3)	0.0200 (18)	0.0020 (17)	0.0095 (15)	0.0014 (19)
C20	0.051 (3)	0.071 (4)	0.024 (2)	0.002 (3)	0.016 (2)	0.010 (2)
C21	0.0173 (18)	0.064 (3)	0.0196 (18)	−0.0011 (18)	0.0084 (15)	−0.012 (2)
C22	0.0131 (16)	0.045 (3)	0.0268 (19)	0.0006 (16)	0.0085 (15)	−0.0130 (18)
C23	0.035 (2)	0.051 (3)	0.037 (2)	−0.003 (2)	0.014 (2)	−0.023 (2)
C24	0.0164 (16)	0.028 (2)	0.0242 (18)	0.0018 (14)	0.0098 (15)	−0.0050 (15)
Cl3	0.0711 (11)	0.1022 (14)	0.0712 (10)	−0.0143 (10)	0.0113 (9)	0.0306 (10)
C25	0.061 (5)	0.052 (5)	0.070 (5)	0	−0.006 (4)	0

### Geometric parameters (Å, º)

**Table:**

Pd1—P1	2.2241 (9)	C12—H12B	0.98
Pd1—Cl2	2.2859 (9)	C12—H12C	0.98
Pd1—Cl1	2.3317 (9)	C13—C14	1.399 (5)
Pd1—Cl1^i^	2.4138 (8)	C13—H13	0.95
Cl1—Pd1^i^	2.4138 (8)	C14—C16	1.387 (5)
P1—C9	1.803 (4)	C14—C15	1.511 (5)
P1—C17	1.809 (3)	C15—H15A	0.98
P1—C1	1.816 (3)	C15—H15B	0.98
C1—C2	1.395 (5)	C15—H15C	0.98
C1—C8	1.397 (5)	C16—H16	0.95
C2—C3	1.398 (5)	C17—C18	1.389 (5)
C2—H2	0.95	C17—C24	1.410 (5)
C3—C5	1.392 (5)	C18—C19	1.404 (5)
C3—C4	1.519 (5)	C18—H18	0.95
C4—H4A	0.98	C19—C21	1.391 (6)
C4—H4B	0.98	C19—C20	1.509 (6)
C4—H4C	0.98	C20—H20A	0.98
C5—C6	1.388 (5)	C20—H20B	0.98
C5—H5	0.95	C20—H20C	0.98
C6—C8	1.391 (5)	C21—C22	1.401 (6)
C6—C7	1.512 (5)	C21—H21	0.95
C7—H7A	0.98	C22—C24	1.389 (5)
C7—H7B	0.98	C22—C23	1.500 (6)
C7—H7C	0.98	C23—H23A	0.98
C8—H8	0.95	C23—H23B	0.98
C9—C10	1.392 (5)	C23—H23C	0.98
C9—C16	1.402 (5)	C24—H24	0.95
C10—C11	1.405 (5)	Cl3—C25	1.755 (5)
C10—H10	0.95	C25—Cl3^ii^	1.755 (5)
C11—C13	1.384 (5)	C25—H25A	0.99
C11—C12	1.502 (5)	C25—H25B	0.99
C12—H12A	0.98		
			
P1—Pd1—Cl2	90.82 (3)	C11—C12—H12C	109.5
P1—Pd1—Cl1	92.10 (3)	H12A—C12—H12C	109.5
Cl2—Pd1—Cl1	173.20 (3)	H12B—C12—H12C	109.5
P1—Pd1—Cl1^i^	173.90 (3)	C11—C13—C14	122.8 (3)
Cl2—Pd1—Cl1^i^	92.18 (3)	C11—C13—H13	118.6
Cl1—Pd1—Cl1^i^	84.35 (3)	C14—C13—H13	118.6
Pd1—Cl1—Pd1^i^	95.65 (3)	C16—C14—C13	117.9 (3)
C9—P1—C17	108.87 (16)	C16—C14—C15	121.1 (3)
C9—P1—C1	108.11 (16)	C13—C14—C15	121.0 (3)
C17—P1—C1	102.52 (15)	C14—C15—H15A	109.5
C9—P1—Pd1	103.29 (11)	C14—C15—H15B	109.5
C17—P1—Pd1	112.19 (12)	H15A—C15—H15B	109.5
C1—P1—Pd1	121.50 (12)	C14—C15—H15C	109.5
C2—C1—C8	120.5 (3)	H15A—C15—H15C	109.5
C2—C1—P1	121.8 (3)	H15B—C15—H15C	109.5
C8—C1—P1	117.4 (3)	C14—C16—C9	120.9 (3)
C1—C2—C3	119.8 (3)	C14—C16—H16	119.5
C1—C2—H2	120.1	C9—C16—H16	119.5
C3—C2—H2	120.1	C18—C17—C24	119.6 (3)
C5—C3—C2	118.3 (3)	C18—C17—P1	122.5 (3)
C5—C3—C4	121.1 (3)	C24—C17—P1	117.7 (3)
C2—C3—C4	120.6 (3)	C17—C18—C19	120.3 (4)
C3—C4—H4A	109.5	C17—C18—H18	119.8
C3—C4—H4B	109.5	C19—C18—H18	119.8
H4A—C4—H4B	109.5	C21—C19—C18	118.8 (4)
C3—C4—H4C	109.5	C21—C19—C20	122.3 (4)
H4A—C4—H4C	109.5	C18—C19—C20	118.9 (4)
H4B—C4—H4C	109.5	C19—C20—H20A	109.5
C6—C5—C3	122.8 (3)	C19—C20—H20B	109.5
C6—C5—H5	118.6	H20A—C20—H20B	109.5
C3—C5—H5	118.6	C19—C20—H20C	109.5
C5—C6—C8	118.1 (3)	H20A—C20—H20C	109.5
C5—C6—C7	121.0 (3)	H20B—C20—H20C	109.5
C8—C6—C7	120.9 (3)	C19—C21—C22	122.1 (4)
C6—C7—H7A	109.5	C19—C21—H21	118.9
C6—C7—H7B	109.5	C22—C21—H21	118.9
H7A—C7—H7B	109.5	C24—C22—C21	118.1 (4)
C6—C7—H7C	109.5	C24—C22—C23	121.0 (4)
H7A—C7—H7C	109.5	C21—C22—C23	120.8 (4)
H7B—C7—H7C	109.5	C22—C23—H23A	109.5
C6—C8—C1	120.4 (3)	C22—C23—H23B	109.5
C6—C8—H8	119.8	H23A—C23—H23B	109.5
C1—C8—H8	119.8	C22—C23—H23C	109.5
C10—C9—C16	119.8 (3)	H23A—C23—H23C	109.5
C10—C9—P1	120.1 (3)	H23B—C23—H23C	109.5
C16—C9—P1	119.4 (3)	C22—C24—C17	121.0 (4)
C9—C10—C11	120.4 (3)	C22—C24—H24	119.5
C9—C10—H10	119.8	C17—C24—H24	119.5
C11—C10—H10	119.8	Cl3—C25—Cl3^ii^	111.8 (5)
C13—C11—C10	118.2 (3)	Cl3—C25—H25A	109.3
C13—C11—C12	122.4 (3)	Cl3^ii^—C25—H25A	109.3
C10—C11—C12	119.5 (3)	Cl3—C25—H25B	109.3
C11—C12—H12A	109.5	Cl3^ii^—C25—H25B	109.3
C11—C12—H12B	109.5	H25A—C25—H25B	107.9
H12A—C12—H12B	109.5		
			
P1—Pd1—Cl1—Pd1^i^	−175.03 (3)	Pd1—P1—C9—C16	−73.6 (3)
Cl1^i^—Pd1—Cl1—Pd1^i^	0	C16—C9—C10—C11	0.6 (5)
Cl2—Pd1—P1—C9	−82.49 (12)	P1—C9—C10—C11	−169.5 (3)
Cl1—Pd1—P1—C9	91.38 (12)	C9—C10—C11—C13	−1.7 (5)
Cl2—Pd1—P1—C17	160.43 (13)	C9—C10—C11—C12	177.0 (3)
Cl1—Pd1—P1—C17	−25.71 (13)	C10—C11—C13—C14	1.4 (5)
Cl2—Pd1—P1—C1	38.80 (13)	C12—C11—C13—C14	−177.2 (3)
Cl1—Pd1—P1—C1	−147.34 (13)	C11—C13—C14—C16	0.0 (5)
C9—P1—C1—C2	134.7 (3)	C11—C13—C14—C15	179.0 (3)
C17—P1—C1—C2	−110.4 (3)	C13—C14—C16—C9	−1.2 (5)
Pd1—P1—C1—C2	15.8 (3)	C15—C14—C16—C9	179.8 (3)
C9—P1—C1—C8	−51.4 (3)	C10—C9—C16—C14	0.9 (5)
C17—P1—C1—C8	63.6 (3)	P1—C9—C16—C14	171.1 (3)
Pd1—P1—C1—C8	−170.3 (2)	C9—P1—C17—C18	21.2 (3)
C8—C1—C2—C3	0.0 (5)	C1—P1—C17—C18	−93.2 (3)
P1—C1—C2—C3	173.7 (3)	Pd1—P1—C17—C18	134.9 (3)
C1—C2—C3—C5	−0.1 (5)	C9—P1—C17—C24	−163.9 (3)
C1—C2—C3—C4	178.6 (4)	C1—P1—C17—C24	81.8 (3)
C2—C3—C5—C6	0.6 (6)	Pd1—P1—C17—C24	−50.2 (3)
C4—C3—C5—C6	−178.1 (4)	C24—C17—C18—C19	1.1 (5)
C3—C5—C6—C8	−0.8 (6)	P1—C17—C18—C19	176.0 (3)
C3—C5—C6—C7	179.0 (4)	C17—C18—C19—C21	−1.6 (5)
C5—C6—C8—C1	0.6 (5)	C17—C18—C19—C20	179.5 (3)
C7—C6—C8—C1	−179.2 (3)	C18—C19—C21—C22	1.0 (5)
C2—C1—C8—C6	−0.2 (5)	C20—C19—C21—C22	179.9 (4)
P1—C1—C8—C6	−174.2 (3)	C19—C21—C22—C24	0.1 (5)
C17—P1—C9—C10	−144.0 (3)	C19—C21—C22—C23	−177.2 (3)
C1—P1—C9—C10	−33.4 (3)	C21—C22—C24—C17	−0.5 (5)
Pd1—P1—C9—C10	96.6 (3)	C23—C22—C24—C17	176.7 (3)
C17—P1—C9—C16	45.8 (3)	C18—C17—C24—C22	−0.1 (5)
C1—P1—C9—C16	156.4 (3)	P1—C17—C24—C22	−175.2 (3)

Symmetry codes: (i) −*x*+1, −*y*+1, −*z*+1; (ii) −*x*+2, *y*, −*z*+3/2.

### Hydrogen-bond geometry (Å, º)

Cg1 and Cg2 are the centroids of rings C17-C19/C21/C22/C24 and C9-C11/C13/C14/C16, respectively.

**Table:**

*D*—H···*A*	*D*—H	H···*A*	*D*···*A*	*D*—H···*A*
C25—H25*A*···Cl2	0.99	2.82	3.733 (4)	154
C21—H21···Cl1^iii^	0.95	2.85	3.693 (4)	148
C5—H5···*Cg*1^iv^	0.95	2.95	3.847 (5)	159
C15—H15*A*···*Cg*2^v^	0.99	2.79	3.620 (5)	143

Symmetry codes: (iii) −*x*+1, *y*, −*z*+1/2; (iv) −*x*+2, −*y*+1, −*z*+1; (v) −*x*+1, −*y*+2, −*z*+1.
